# Psychosocial Health and Quality of Life Among Living Kidney Donors: A Study From a Tertiary Care Center in South India

**DOI:** 10.1016/j.xkme.2026.101361

**Published:** 2026-04-13

**Authors:** Anjana Gopal, Rajesh R. Nair, George Kurian, Anil Mathew, Zachariah Paul, P. Sivakami, K.P. Lakshmi

**Affiliations:** 1 Department of Nephrology, Amrita Institute of Medical Sciences, Kerala, India; 2 Department of Psychiatry, Amrita Institute of Medical Sciences, Kerala, India

**Keywords:** Anxiety, depression, living donor, quality of life

## Abstract

**Rationale & Objectives:**

Although kidney donation has been deemed a safe procedure with low mortality and morbidity, the impact of organ donation on the long-term quality of life is not known in our population. We aimed to compare the quality of life between age and sex-matched living kidney donors and healthy nondonors and to estimate the prevalence of anxiety, depression, and regret among kidney donors.

**Study Design:**

Questionnaire-based observational study.

**Setting & Participants:**

Adult living kidney donors aged ≥18 years who donated between 2020 and 2024 at a tertiary care center in South India.

**Predictors:**

We analyzed the effects of baseline clinical, sociodemographic factors, postoperative complications, long-term comorbid conditions, and recipient and donor renal function on the psychosocial health of living kidney donors.

**Outcomes:**

Quality of life and the prevalence of anxiety, and depression were determined using the SF-36 (36-Item Short Form Health Survey), General Anxiety Disorder-7 (GAD-7), and Patient Health Questionnaire-9 (PHQ-9), respectively.

**Results:**

Of the 69 included living kidney donors, 57 (82.6%) were female. Post-donation anxiety and depression were seen in 7 (10.1%) donors and 3 (4.3%) donors, respectively. The SF-36 Mental Component Summary and Physical Component Summary scores of donors were not statistically different from those of age- and sex-matched healthy donors (46.84 ± 8.72 vs 48.48 ± 7.52; *P* = 0.24 and 46.42 ± 9.25 vs 47.24 ± 12.02; *P* = 0.66, respectively). Further age stratification revealed that donors older than 50 years performed poorly in various domains of the SF-36 compared with younger donors. The donors who developed medical comorbid conditions, surgical complications, or deconditioning limiting physical activity post-donation were unlikely to recommend others to donate.

**Limitations:**

The study was conducted at a single center with a relatively small sample size.

**Conclusions:**

The quality of life of kidney donors was comparable to that of healthy nondonors except for a subgroup of donors older than 50 years who had worse quality of life parameters in certain domains compared with younger donors. Overall, living kidney donors reported a positive donation experience, with low rates of regret.

## Plain-language Summary

Living kidney donation is generally considered safe, but its long-term effects on donors’ mental well-being and daily life are not well studied. This study examined the quality of life, anxiety, depression, and regret among people who donated a kidney at a tertiary hospital in South India and compared them with healthy nondonors of similar age and sex. Most donors reported a positive donation experience, good kidney health, and no regret. Levels of anxiety and depression were low. Overall quality of life was comparable between donors and nondonors. However, donors older than 50 years of age reported more physical pain, lower physical functioning, and reduced emotional well-being. These findings highlight the need for targeted medical and psychological follow-up, especially for older kidney donors.

According to the annual report of the National Organ and Tissue Transplant Organization, a total of 13,426 kidney transplants were completed in 2023, of which 11,791 were living donor kidney transplants.^1^ To improve the recipient’s quality of life, the donor endures short- and long-term health risks^2^ without any personal health benefits. Although donor surgery has been deemed a safe procedure with low mortality and morbidity,^3^, ^4^, ^5^ the impact of organ donation on long-term psychosocial health and quality of life is not well studied in our population. Considering that in India, more than 85% of kidney transplants are from living kidney donors, there are few studies on the psychosocial health and quality of life of living kidney donors from India.

During follow-up of kidney donors, we typically focus on assessing surgical and medical outcomes but often overlook psychosocial aspects. However, the prevalence of post-donation psychosocial adverse outcomes is not negligible.^6^, ^7^, ^8^ A detailed understanding of the potential psychosocial benefits and harms of kidney donation can help us improve the informed consent process and counseling during the donation process.

Most of the prior studies were retrospective analyses exploring quality of life; there are very few studies comparing donors and nondonors or studies analyzing risk factors for poor outcomes.^9^, ^10^, ^11^ Previous studies from India have shown that quality of life was improved post-donation, and the prevalence of depression and anxiety was low.^9^, ^10^, ^11^ However, a comparative analysis of quality of life among age- and sex-matched donors and nondonors from a similar socioeconomic background is lacking; such an analysis would help us better understand the psychosocial implications of donation and the risk factors contributing to it. A more precise definition of these risk factors may guide the implementation of interventions to prevent negative consequences and thus improve the information provided to potential donors during the pre-donation assessment. The prevalence of depression and anxiety is also unclear, with wide ranges reported in the literature, ranging from 0% to 46.9% and 0% to 67%, respectively.^12^ Our study aimed to compare the Quality of life between living kidney donors and healthy nondonors and analyzed the prevalence of anxiety, depression and regret among kidney donors.

## Methods

This was a retrospective questionnaire-based study conducted among living kidney transplant donors (aged ≥18 years) who underwent kidney donation at the Amrita Institute of Medical Sciences between 2021 and 2024. The study was performed according to the guidelines in the Declaration of Helsinki, and the Institute’s Internal Ethics Committee (Amrita Institute of Medical Sciences, Kochi) reviewed and approved the study protocol (IEC-AIMS-2025-NEPHRO-002). Consent for participation in the study and for publication of clinical details and images was obtained. Participants understood that names and initials would not be published, and all standard protocols would be followed to conceal their identity.

As per institution protocol, all donors undergo a pre-donation psychiatric evaluation. Those who offered to participate in the study were declared free of morbid depression and anxiety. They were also not taking any medicine or undergoing counseling sessions. Donors also undergo extensive psychosocial evaluation by a social worker to ensure that the donation is voluntary and free of coercion.

A comparison group of age and sex-matched healthy nondonors from a similar socioeconomic background was recruited from among the healthy family members of the donors. They were eligible for inclusion in the study if they were healthy (ie, without renal disease, hypertension, diabetes, cardiovascular disease, pulmonary disease, psychiatric illness, or active cancer, as reported by historical recall), were at least 18 years of age, and were proficient in the native language and able to complete the questionnaires.

### Study procedure

The participants were interviewed either in person or via phone/video call by the research personnel. Baseline demographic and clinical data were collected from medical records and interviews. Details about the donor’s experience in the posttransplant recovery period, motivation, interpersonal relationship with the recipient, and long-term outcomes were also collected.

For the assessment of quality of life, the 36-Item Short Form Health Survey (SF-36) was used. This tool has been translated into Malayalam and validated in previous studies.^13^ It measures quality of life based on 8 dimensions. Raw scores range from 0 to 100, with higher scores indicating better quality of life. Physical and mental component summary scores were also generated. The Physical Component Summary is an aggregate score of the physical functioning, physical role, bodily pain, and general health domains of the SF-36. The Mental Component Summary is an aggregate score of the energy/vitality, social functioning, emotional role, and mental health domains.

For the assessment of anxiety and depression, we used the General Anxiety Disorder-7 (GAD-7) and Patient Health Questionnaire-9 (PHQ-9) scales, respectively. The GAD-7 questionnaire is a self-administered tool for assessing generalized anxiety disorder and anxiety-related symptoms. Each question response category—‘not at all,’ ‘several days,’ ‘more than half the days,’ and ‘nearly every day’—is assigned a score of 0, 1, 2 and 3, respectively. The total score is calculated by adding together the individual scores for the 7 questions. Final scores of 5, 10, and 15 are the cutoffs for mild, moderate, and severe anxiety, respectively. We defined post-donation anxiety as a score of ≥9 on the GAD-7.^14^^,^^15^

The PHQ-9 questionnaire contains 9 items, including questions on loss of interest, depressed mood, sleep, appetite and energy changes, low self-worth, difficulty concentrating, psychomotor activity changes, and suicidal ideation. For each item, responses are scored between 0 and 3, ranging from ‘not at all’ to ‘nearly every day’; overall scores range from 0 to 27. The construct validity and criterion validity have been tested in general populations, with a PHQ-9 score ≥10 found to have 88% sensitivity and 88% specificity for clinical depression.^16^ Scores of 5, 10, 15, and 20 represent cutoffs for mild, moderate, moderately severe, and severe depression. We defined post-donation depression as a score of ≥10 on the PHQ-9 depression questionnaire.^17^

### Statistical Analysis

Descriptive analysis results are presented as mean ± standard deviation for continuous variables and percentages for categorical variables. To analyze the association between groups and SF-36 scores, bivariate analysis was performed using *t* tests or Mann-Whitney *U* tests, as appropriate based on the distribution of the variables. Bivariate analyses using *t* tests, Mann-Whitney *U* tests, and χ^2^ tests were done to identify risk factors having a significant association with impaired quality of life domains in SF-36. *P* < 0.05 was considered statistically significant.

## Results

Between 2021 and 2024, approximately 140 living donor kidney transplants were performed at our center. Of these, 71 donors (50.7%) either declined participation or could not be contacted, resulting in a final study cohort of 69 donors. The most common reasons cited were lack of time, travel constraints, and reluctance to complete psychological questionnaires, rather than dissatisfaction with the donation process. Among the participants, 57 (82.6%) were female, with a mean age of 50.78 ± 9.50 years. Based on the Modified Kuppuswamy scale, 60.9% belonged to the lower-middle socioeconomic class. The donor–recipient relationship distribution was as follows: mothers (34.8%), wives (33.3%), siblings (13.0%), fathers (10.1%), husbands (4.3%), and others (4.3%). Pretransplant comorbid conditions included hypertension in 2.9% and dyslipidemia in 1.45% of donors (Table 1).

**Table 1:** Baseline Characteristics of Study Participants (N = 69)

Characteristic	Value
Age, y, mean ± SD	50.78 ± 9.50
Sex	
Male	12 (17.4%)
Female	57 (82.6%)
Married	69 (100%)
Kuppuswamy scale	
Upper class	—
Upper middle	20 (29%)
Lower middle	42 (60.9%)
Upper lower	7 (10.1%)
Lower	—
Employed	33 (47.8%)
Educated above high school	54 (78.3%)
Relation	
Wife	23 (33.3%)
Husband	3 (4.3%)
Mother	24 (34.8%)
Father	7 (10.1%)
Brother	2 (2.9%)
Sister	7 (10.1%)
Grandmother	2 (2.9%)
Aunt	1 (1.4%)
Time since donation, mo, mean ± SD	30.38 ± 16.96
Pretransplant hypertension	
Yes	2 (2.9%)
No	67 (97.1%)
Pretransplant dyslipidemia	
Yes	1 (1.4%)
No	68 (98.6%)
Pretransplant hypothyroidism	
Yes	7 (10.1%)
No	62 (89.9%)

*Note:* Values are n (%) unless otherwise indicated.

Abbreviation: SD, standard deviation.

The mean duration since donation was 30.38 ± 16.96 months, with a mean follow-up serum creatinine level of 1.05 ± 0.22 mg/dL. All donors reported that their decision to donate was voluntary, and none expressed regret. Furthermore, all felt adequately informed about the donation process, and no donor described an unpleasant post-donation experience. However, prolonged post-donation pain exceeding expectations was reported by 62.3% of participants. Poor appetite and disturbed sleep were each reported by 2.9% of donors. Based on the GAD-7 and PHQ-9 questionnaires, post-donation anxiety and depression were identified in 7 (10.1%) and 3 (4.3%) donors, respectively (Table 2).

**Table 2:** Post-Donation Outcomes

Outcome	Value
Diabetes mellitus	
Yes	2 (2.9%)
No	67 (97.1%)
Hypertension -n (%)	
Yes	9 (13%)
No	60 (87%)
Dyslipidemia -n (%)	
Yes	5 (7.2%)
No	64 (92.8%)
Hypothyroidism -n (%)	
Yes	4 (5.8%)
No	65 (94.2%)
Coronary artery disease -n (%)	
Yes	2 (2.9%)
No	67 (97.1%)
Cerebrovascular accident-n (%)	
Yes	3 (4.3%)
No	66 (95.7%)
Chronic liver disease	
Yes	1 (1.4%)
No	68 (98.6%)
Creatinine at last follow-up, mg/dL, mean ± SD	1.05 ± 0.22
Recipient graft function	
Graft loss	2 (2.9%)
Good graft function	66 (95.7%)
Died	1 (1.4%)
Postoperative complication	
Prolonged pain	43 (62.3%)
Poor appetite	2 (2.9%)
Poor sleep	2 (2.9%)
Prolonged hospital stay	2 (2.9%)

*Note:* Values are n (%) unless otherwise indicated.

Abbreviation: SD, standard deviation.

Thirteen donors (18.8%) reported an improvement in their relationship with the recipient after donation, indicating a stronger bond, whereas the remaining 81.2% perceived no change. When asked about their likelihood of recommending kidney donation to others, 6 donors (8.7%) responded ‘very likely,’ 54 (78.2%) responded ‘likely,’ 4 (5.7%) patients were ‘neutral,’ and 5 (7.2%) responded ‘unlikely.’ In a detailed interview of participants who were unlikely to recommend donation, donors who developed post-donation medical comorbid conditions (such as diabetes mellitus or cardiovascular disease), surgical complications (re-exploration/wound infection) or prolonged pain and deconditioning interfering with routine daily activity gave a negative response.

**Figure 1:**
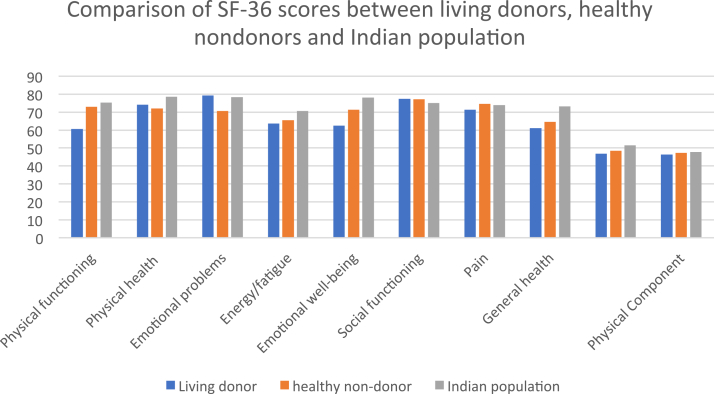
Comparison of 36-Item Short Form Health Survey (SF-36) scores between living donors, healthy nondonors, and the Indian population.

A comparison of SF-36 scores between living donors and age- and sex-matched healthy controls is presented in Table 3. Figure 1 illustrates these scores alongside reference values from Indian population data.^18^ Living donors scored significantly lower than healthy controls in physical functioning (60.65 ± 23.74 vs 72.97 ± 26.30; *P* = 0.005) and emotional well-being (62.37 ± 23.45 vs 71.35 ± 17.36; *P* = 0.01). Physical functioning reflects limitations in physical activity due to health conditions (eg, walking, climbing stairs, lifting), while emotional well-being assesses the impact of emotional problems on daily activities. Univariate analysis revealed that age >50 years (*P* = 0.03) was the only significant predictor of poor performance in the emotional well-being domains. Univariate analysis did not reveal any significant predictors of poor performance in the other domains.

**Table 3:** Comparison of SF-36 Domains Between Living Donors and Healthy Nondonors

Variable	Living Donors (N = 69)	Healthy Nondonors (N = 69)	P
Age, y	50.78 ± 9.508	47.75 ± 11.705	0.10
Sex, n (%)			
Male	12 (37.5%)	20 (62.5%)	0.11
Female	57 (53.8%)	49 (46.2%)	
SF-36 domain			
Physical functioning	60.65 ± 23.747	72.97 ± 26.309	0.005^a^
Role limitation due to physical health	74.15 ± 32.93	72.10 ± 41.46	0.75
Emotional problems	79.22 ± 34.82	70.76 ± 43.20	0.21
Energy/fatigue	63.62 ± 21.96	65.43 ± 14.13	0.57
Emotional well-being	62.37 ± 23.45	71.35 ± 17.36	0.01^a^
Social functioning	77.35 ± 23.41	77.17 ± 23.67	0.96
Pain	71.44 ± 22.82	74.67 ± 23.70	0.42
General health	61.15 ± 20.20	64.56 ± 19.66	0.32
Mental Component Summary score	46.84 ± 8.72	48.48 ± 7.52	0.24
Physical Component Summary score	46.42 ± 9.25	47.24 ± 12.02	0.66

*Note:* Values are mean ± standard deviation unless otherwise indicated.

Abbreviation: SF-36, 36-Item Short Form Health Survey.

a Statistically significant.

A subgroup analysis was performed to assess the impact of age on quality of life domains (Table 4). When we compared baseline donor characteristics by age, the only statistically significant differences were in educational status (*P* = 0.02) and employment status (*P* = 0.06). Donors aged ≤50 years had significantly higher scores in social functioning (*P* = 0.02), bodily pain (*P* = 0.04), and the Physical Component Summary (*P* = 0.03) compared with older donors. In all domains except general health, mean scores were numerically higher in the younger age group. In the comparison of the SF-36 quality of life domain scores between donors and nondonors aged >50 years, statistically significant differences were observed in the physical functioning (56.94 ± 25.05 vs 70 ± 26.60; *P* = 0.05) and emotional well-being (60.00 ± 25.08 vs 71.68 ± 18.57; *P* = 0.05) domains.

**Table 4:** Comparison of SF-36 Domains Between Donors Aged ≤50 Years and >50 years

Variable	Aged ≤50 y (N = 33)	Aged >50 y (N = 36)	*P*
SF-36 domain			
Physical functioning	64.70 ± 21.900	56.94 ± 25.051	0.18
Role limitation due to physical health	80.30 ± 29.81	68.51 ± 35.023	0.14
Emotional problems	86.86 ± 27.56	72.22 ± 39.44	0.08
Energy/fatigue	68.18 ± 19.99	59.44 ± 23.10	0.10
Emotional well-being	64.97 ± 21.63	60.00 ± 25.08	0.38
Social functioning	84.09 ± 18.55	71.18 ± 25.84	0.02^a^
Pain	77.34 ± 17.51	66.04 ± 25.85	0.04^a^
General health	60.45 ± 19.13	61.81 ± 21.38	0.78
Mental Component Summary score	47.38 ± 7.73	46.34 ± 9.62	0.62
Physical Component Summary score	49.00 ± 7.85	44.07 ± 9.90	0.03^a^

*Note:* Values are mean ± standard deviation.

Abbreviation: SF-36, 36-Item Short Form Health Survey.

a Statistically significant.

## Discussion

Our study reveals that the majority of the living kidney donors had a positive donor experience and did not regret their decision to donate their kidney. Apart from prolonged post-donation pain—which exceeded preoperative expectations—no major medical complications were observed. The preservation of excellent kidney function in our cohort is reassuring and likely reflects the stringent donor selection criteria implemented at our center. In addition to clinical outcomes, our study also examined psychosocial well-being, an aspect often underrepresented in post-donation assessments. Post-donation anxiety and depression were observed in 7 (10.1%) and 3 (4.3%) donors, respectively—rates consistent with prior reports indicating low prevalence of anxiety (5.5%), depression (4.2%), and regret (2.1%) among living kidney donors.^9^^,^^19^^,^^20^

To our knowledge, this is the first study to compare quality of life between age- and sex-matched donors from comparable socioeconomic backgrounds. In contrast, prior research has predominantly employed observational designs or prospective within-group comparisons.

Quality of life assessment revealed lower scores among donors than healthy nondonors in 2 SF-36 domains—physical functioning (60.65 ± 23.74 vs 72.97 ± 26.30; *P* = 0.005) and emotional well-being (62.37 ± 23.45 vs 71.35 ± 17.36; *P* = 0.01)—while overall Physical Component Summary and Mental Component Summary scores were comparable between groups. These findings align with systematic review evidence suggesting that quality of life following donation is generally preserved or improved, with very low rates of regret.^21^

Although some studies have reported superior SF-36 scores in donors compared with controls,^22^, ^23^, ^24^, ^25^ others have documented modest declines in certain domains.^26^^,^^27^ Several prospective studies of living kidney donors, comparing pre- and post-donation status, have even shown an improvement in quality of life.^11^^,^^28^ However, some studies did report higher bodily pain compared with the general population.^29^^,^^30^ In the ELIPSY study, a subgroup of donors suffered a slight deterioration in some aspects of their quality of life, and the major contributing factor was attributed to insufficient preoperative risk disclosure.^31^ Similarly, prior studies exploring the risk factors associated with poor quality of life have identified the following as contributory: poor recipient outcomes,^32^^,^^33^ donation-related complications,^34^ a history of psychiatric symptoms, poorer physical health pre-donation, a longer recovery time post-donation, greater financial burden, being poorly informed about donation,^35^ and medical complications for either the donor or the transplant recipient.^36^, ^37^, ^38^ However, in our univariate analysis, the only significant factor contributing to poor outcomes was age >50 years. We were unable to identify any other reason for low performance in certain domains, which could be attributed to our small sample size.

Subgroup analysis in our cohort revealed that donors aged >50 years scored lower across the bodily pain and social functioning SF-36 domains. Even when compared with nondonors in this age category, donors performed poorly in these domains. This is contrary to what was shown in previous studies on the quality of life of older donors.^11^^,^^39^, ^40^, ^41^, ^42^ Our in-depth interviews with these participants revealed that the development of medical comorbid conditions, fatigue, and physical deconditioning after donation played a role in their comparatively lower scores in these domains. Post-donation fatigue^43^, ^44^, ^45^, ^46^, ^47^, ^48^ and prolonged pain^7^^,^^49^, ^50^, ^51^, ^52^ have also been documented in previous studies and identified as significant factors contributing to diminished quality of life. Fatigue can contribute to a decline in physical function and social engagement and delayed return to work, which can indirectly lead to a poor quality of life. Considering that the majority of the donors were parents (especially those in the older than 50 years subgroup), we expected them to exhibit superior emotional well-being and quality of life, but our findings were quite the contrary. We hypothesize that Indian parents, as primary caregivers, assume significant responsibilities in the posttransplant care of recipients, often at the expense of their own health. Additionally, their role as primary income earners may hinder their ability to resume employment, compounding the psychosocial and economic burden. These compounded stressors may contribute to the adverse psychosocial outcomes observed in this subgroup. Consistent with our findings, previous studies have also reported that parental donors frequently experience higher levels of post-donation pain and fatigue than initially anticipated.^53^

These findings underscore the importance of targeted follow-up strategies, including structured medical and psychosocial support, for at-risk donors, with a focus on post-donation rehabilitation and counseling. Further studies focusing on in-depth interviews with the subgroup experiencing poor outcomes are needed to better understand the risk factors, as well as focused group discussions to develop strategies for mitigating their adverse outcomes. Overall, our results are reassuring, indicating that living kidney donation does not have an adverse effect on overall quality of life.

### Limitations

This study has several limitations. The study was conducted at a single center with a relatively small sample size, which limits its generalizability. Nonresponse bias is a concern, as nearly half of the donors did not complete the questionnaire, potentially omitting perspectives that may have influenced results. In addition, the interview- and questionnaire-based design introduces the possibility of recall bias. We also acknowledge the potential confounding effects of socioeconomic stressors and the inability of cross-sectional instruments like the GAD-7 and PHQ-9 to distinguish donation-related distress from broader life circumstances. Future multicenter prospective studies with higher response rates are needed to validate these findings and to better characterize long-term psychosocial outcomes, particularly in older donor populations.

### Conclusions

Overall, living kidney donors reported a positive donation experience, with low rates of regret and a comparable quality of life to that of nondonors. Our study revealed that a subgroup of donors older than 50 years had worse SF-36 quality of life scores in certain domains compared with younger donors. These findings underscore the importance of providing targeted post-donation medical and psychosocial support, particularly for older donors, to optimize long-term outcomes.
